# A useful approach to sensitivity and predictability studies in geophysical fluid dynamics: conditional non-linear optimal perturbation

**DOI:** 10.1093/nsr/nwz039

**Published:** 2019-03-19

**Authors:** Qiang Wang, Mu Mu, Guodong Sun

**Affiliations:** 1 CAS Key Laboratory of Ocean Circulation and Waves, Institute of Oceanology, Chinese Academy of Sciences, Qingdao 266071, China; 2 Pilot National Laboratory for Marine Science and Technology (Qingdao), Qingdao 266237, China; 3 Center for Ocean Mega-Science, Chinese Academy of Sciences, Qingdao 266071, China; 4 Department of Atmospheric and Oceanic Sciences, Institute of Atmospheric Sciences, Fudan University, Shanghai 200438, China; 5 LASG, Institute of Atmospheric Physics, Chinese Academy of Sciences, Beijing 100029, China; 6 University of Chinese Academy of Sciences, Beijing 100049, China

**Keywords:** CNOP, non-linear optimization, atmosphere, ocean

## Abstract

In atmospheric and oceanic studies, it is important to investigate the uncertainty of model solutions. The conditional non-linear optimal perturbation (CNOP) method is useful for addressing the uncertainty. This paper reviews the development of the CNOP method and its computational aspects in recent years. Specifically, the CNOP method was first proposed to investigate the effects of the optimal initial perturbation on atmosphere and ocean model results. Then, it was extended to explore the influences of the optimal parameter perturbation, model tendency perturbation and boundary condition perturbation. To obtain solutions to these optimal perturbations, four kinds of optimization approaches were developed: the adjoint-based method, the adjoint-free method, the intelligent optimization method and the unconstrained optimization method. We illustrate the calculation process of each method and its advantages and disadvantages. Then, taking the Zebiak–Cane model as an example, we compare the CNOPs related to initial conditions (CNOP-Is) calculated by the above four methods. It was found that the dominant structures of the CNOP-Is for different methods are similar, although some differences in details exist. Finally, we discuss the necessity and possible direction for designing a more effective optimization approach related to the CNOP in the future.

## INTRODUCTION

Numerical models have become indispensable tools in geophysical fluid dynamics research. However, as is well known, uncertainties exist in both the initial conditions and the models themselves due to inadequate observations, unresolved physical processes, numerical truncation errors and so on. These uncertainties can lead to errors in the model solutions [1,2]. Sensitivity and predictability studies are just to investigate how the initial condition and model uncertainties result in the uncertainties of model solutions, to reveal the reasons and mechanisms and explore approaches to reduce the uncertainties in model solutions [3–5].

Sensitivity and predictability problems can be generally divided into two categories: one related to the uncertainty of initial conditions under the assumption of a perfect model and the other related to model uncertainty with a perfect initial field [6]. These problems are related to the stability of fluid dynamical fields such as those in atmospheric and oceanic flows. Therefore, the well-known tool for fluid dynamical stability analysis, normal mode [7,8], has been adopted to address these problems [9–11]. However, [12,13] pointed out that the transient growth of perturbations can still occur in the absence of growing normal modes, which implies that the normal modes cannot well represent the unstable modes of the atmosphere and ocean. Therefore, the normal mode theory might not be able to deal well with the sensitivity and predictability problems in geophysical fluid dynamics.

The singular vector (SV), which is also called non-normal mode, has been employed to address the above problems. For example, in an atmospheric study, [14] used the SV method to examine the weather predictability; in an oceanic study, [15] and [16] employed the approach to investigate the predictability of the Atlantic meridional overturning circulation and the Kuroshio path variations, respectively. The SV approach has also been used to assess the effect of the model error on the solutions [17]. More applications of the SV method have been summarized in [18].

Notably, both the normal mode and the SV approach are based on the assumption of linear approximation, which demands that the perturbation is sufficiently small and that the evolution of the perturbation can be approximately represented by a tangent linear model. In this case, the impact of non-linear physical processes cannot be well considered. To overcome this limitation, Mu proposed the novel method of the non-linear singular vector (NSV) and the non-linear singular value (NSVA) in [19]. Subsequently, [20] calculated the NSV and NSVA within a 2D quasi-geotropic model and found that local fastest-growing perturbations can be obtained under some basic flows for which the objective function attains the local maximum, but such a point could play important roles in sensitivity and predictability studies. Hence, to study the sensitivity and predictability problem, it is necessary to determine all local fastest-growing perturbations, which is very inconvenient in realistic applications.

To overcome the disadvantage of NSV, in 2003, Mu and his collaborators proposed an innovative method to explore the optimal perturbation that can fully consider the non-linear effect without any linear approximation assumption by using a non-linear optimization method [21], which is called the conditional non-linear optimal perturbation (CNOP). It is interesting to point out that, in ref. [22], when Mu and the coauthors studied the predictability problems in weather and climate, they aimed to estimate the upper bound of prediction error by using the information of initial and model parameter uncertainties. A non-linear constraint optimization problem is formulated for this purpose (see [22], Equation (2.7)). The solution to yield the upper bound is nothing but the CNOP, despite this terminology not being introduced there. It is also worthwhile to mention that the CNOP approach was reported to solve the stability and sensitivity problem of geophysical fluid dynamics in the 16th Australasian Fluid Mechanics Conference in 2007 [23]. Note that, in the fluid mechanics field, an optimal initial perturbation method that is exactly the same as the CNOP approach was reported to investigate the transition from the laminar state to the turbulent state in 2010 (see [24,25]).

Physically, the CNOP represents the initial perturbation that has maximal non-linear evolution under a given physical constraint. Based on this, the CNOP can be applied to not only explore the optimal precursor signal of an atmosphere and ocean anomaly event and thus reveal the mechanism of the occurrence of the event, but can also be used to investigate the fastest-growing initial error in the prediction of the event and the related predictability property.

The CNOP approach mentioned above is related to the initial perturbation. In recent years, the approach has been extended to explore the effects of model parameter errors, model tendency errors and boundary condition errors [26–28]. Simultaneously, the approach has been applied to investigate the stability of the thermohaline circulation [29,30], the El Niño Southern Oscillation (ENSO) predictability [31,32], the predictability of the Kuroshio path variations [33], typhoon-targeted observations [34,35], the parameter sensitivity of the land surface processes [36] and others. These applications indicate that the CNOP approach is a useful tool for studying the sensitivity and predictability of the atmosphere and ocean.

A key step when applying the CNOP approach is how to obtain the CNOP numerically because it is almost impossible to have an analytical solution to such a non-linear complicated optimization problem of high dimensions. In recent years, different algorithms, such as spectral projected gradient (SPG) [37] and sequential quadratic programming (SQP) [38], have been adopted to obtain the CNOP. Simultaneously, some new algorithms have also been developed. In these algorithms, the most direct one is the adjoint-based algorithm, which needs to use the tangent linear model of numerical model and its adjoint model. Refs. [21,39] employed this kind of algorithm to calculate the CNOP. However, the adjoint model for some numerical models is not available. Therefore, some adjoint-free computational approaches, such as the ensemble-based method [40] and the intelligent optimization method [41], have been developed to compute the CNOP. Of course, as the atmosphere and ocean models become more complex and the resolution becomes finer, some challenges for the CNOP computation remain.

To exhibit the development of the CNOP computational method, the progress in the CNOP applications and to highlight its new application potential, this review will first introduce the CNOP approach and then systematically present the development of the CNOP calculation method and related applications. Finally, computational challenges in future applications of the CNOP will be discussed.

## CNOP APPROACH

As mentioned above, the CNOP approach was first proposed to explore the impact of initial error on the model results and was then extended to investigate the influences of model parameter errors, model tendency errors and boundary condition errors. Hence, this section will introduce the CNOP method in a uniform form following [42].

Generally, an atmosphere and ocean model can be formally written as 
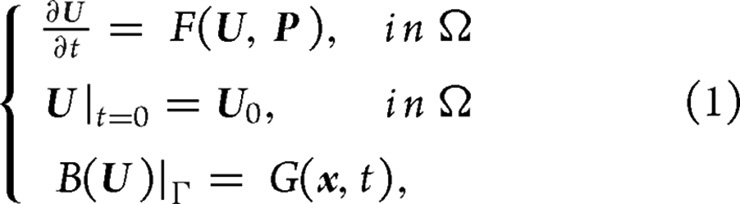


where ${\boldsymbol{U}}$denotes the model state vector, ${\boldsymbol{P}}$is the model parameter vector, $F$is the non-linear operator; ${\boldsymbol{x}} \in {\rm{\Omega \!}}$, where${\rm{\ \Omega }}$is a region, ${\rm{\Gamma }}$is the boundary of${\rm{\ \Omega }}$, $B$is the boundary condition operator, and ${{\boldsymbol{U\!}}_0}$ and $G$represent the initial condition and boundary condition, respectively.

Assume that the initial perturbation ${{\boldsymbol{u\!}}_0}$, boundary condition perturbation $g( {{\boldsymbol{x\!}},\ t\!} )$, model parameter perturbation ${\boldsymbol{p}}( t )$ and model tendency perturbation $f( {{\boldsymbol{x\!}},\ t} )$ exist; Equation (1) then becomes 
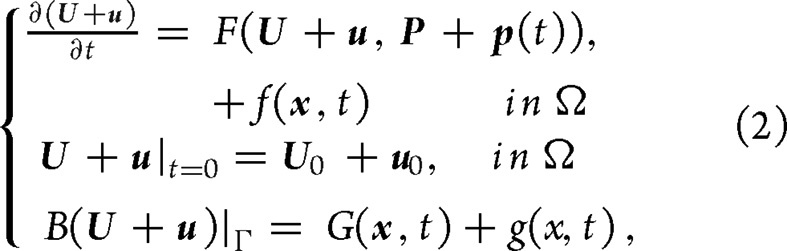


where ${\boldsymbol{u}}$ denotes the difference from the reference state ${\boldsymbol{U}}$ caused by the perturbations in the initial condition, boundary condition, model parameters and model tendency. To assess the maximal influences of the perturbations, the following optimization problem is defined as

(3) \begin{eqnarray*} &&J\!\!\! \left( {{{\boldsymbol{u}}_{0\delta }},{\rm{\ }}{{\boldsymbol{p}}_\varepsilon\! }\left( t\! \right),{f_\gamma\! }\left( {{\boldsymbol{x}},t} \right),{\boldsymbol{\ }}{g_\sigma }\left( {{\boldsymbol{x}},t} \right)} \right)\nonumber\\ &&=\max J\!\!\!\left( {{\boldsymbol{u}}\left( \tau \right)} \right),\end{eqnarray*}

where $J$is an objective function that can be defined according to the considered physical problem. Generally, the perturbation amplitudes are limited, which leads to the perturbations satisfying the constraint condition ${{\boldsymbol{u}}_0} \in {C_\delta\!\!}$, ${\boldsymbol{p}}( t ) \in {C_\varepsilon\!\!}$, $f( {{\boldsymbol{x\!}},\ t} ) \in {C_\gamma }$and $g( {{\boldsymbol{x\!}},\ t} ) \in {C_\sigma\!\!}$. This constraint condition can be simply defined as a ball constraint, or a functional set that satisfies certain conditions. The solution of the above optimization problem $( {{{\boldsymbol{u}}_{0\delta }},{\rm{\ }}{{\boldsymbol{p}}_\varepsilon\! }( t ),{\boldsymbol{\ }}{f_\gamma\!}( {{\boldsymbol{x\!}},\ t} ),{\boldsymbol{\ }}{g_\sigma }( {{\boldsymbol{x\!}},\ t} )} )$is called the CNOP, which represents the optimal combined mode that consists of the perturbations in initial condition, parameters, tendency and boundary condition.

Interestingly, if we only consider the initial condition perturbation (i.e. the other perturbations are set to 0), the solution of Equation (3) becomes the CNOP related to the initial condition, abbreviated as CNOP-I; that is, the CNOP-I proposed by [21] is a specific case of the CNOP. Similarly, we can also define the other special cases such as the CNOP related to parameter (CNOP-P), tendency or forcing (CNOP-F) and boundary condition (CNOP-B).

It is worth mentioning that, from the view of mathematics, the CNOP is an application of the non-linear optimization to the atmosphere and ocean sciences. The other important application is the 4D variational (4-D Var) assimilation. However, the 4-D Var assimilation is to solve a *minimum* problem, which is used to look for the optimal (*best*) initial condition and values of model parameters for the model simulation and prediction. But the CNOP method is to solve a *maximum* problem; it seeks the optimal perturbation that has the *maximal* effect on the model simulation and prediction. This means that the CNOP approach is different from 4-D Var assimilation in principle.

## CNOP COMPUTATION

Currently, different optimization approaches have been developed to obtain a CNOP by solving the optimization Equation (3). In the following, we will review these approaches, including the adjoint-based and adjoint-free methods, the intelligent optimization method and the unconstrained optimization method.

### Adjoint-based optimization method

A key step to solve the optimization Equation (3) is to find the increasing direction of the objective function value. Generally, this direction can be determined by the gradient of the objective function. As derived in refs. [26–28], the most direct way to obtain the gradient is to integrate the adjoint model associated with the considered atmosphere and ocean model.

Errico systematically introduced the adjoint model and its application to the study of the atmosphere and ocean sciences [43]. As is well known, running an atmosphere and ocean model means integrating the model *forward* in time. However, the adjoint model is integrated *backward* in time. This kind of backward integral can give the gradient information of the objective function.

**Figure 1.**
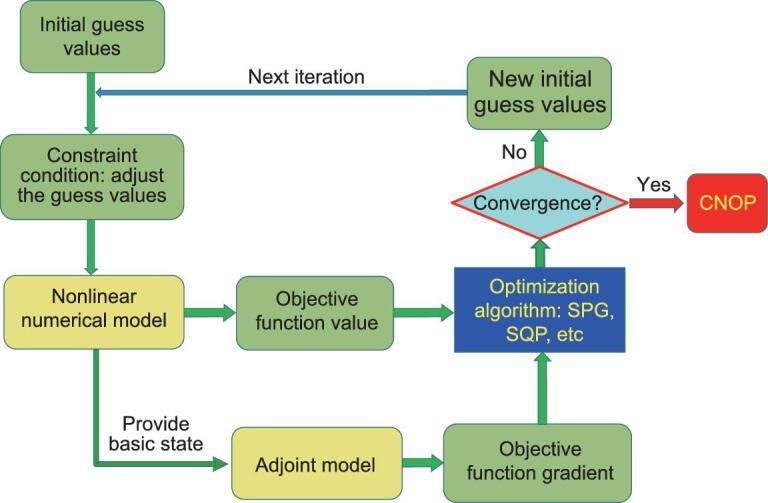
Schematic diagram of the adjoint-based method for calculating the CNOP.

During real calculations, the objective function value needs to be computed in addition to the objective function gradient. The non-linear numerical model can be used to calculate the function value. Then, this value and the gradient information are provided to the optimization algorithm to obtain the CNOP, as shown in Fig. 1. Currently, the adjoint method for computing the CNOP has been applied to investigate the ENSO predictability [44–48], the thermohaline circulation stability [29,30], the stability of the ocean double-gyre circulation [49], the Kuroshio predictability [33,39,50] and the typhoon-targeted observation [34,35], among others. The dimensions of the optimization problem in these applications varies from *Ο*(1) to *Ο*(10^7^), as shown in Table 1. The maximal dimension is approximately 1.71 × 10^7^ in ref. [39].

**Table 1.** The summary of different methods used to obtain the CNOP. Dimension of the optimization problem in the third column can be estimated by the sum of the total grid number in the horizontal and vertical directions for each optimized variable.

Model	Optimization algorithm	Dimension of the optimization problem	Dimension reduction	Dimension of post-treatment of the optimization problem	Reference
ZC model [53]	SPG [37] and adjoint-based method	1080	No	×	[46] (2013)
	SPG and adjoint-free method	1080	Yes	10∼100	[62] (2015)
	Cooperative co-evolution-based particle swarm optimization (CCPSO) algorithm	270	No	×	[76] (2015)
	PCA-based PSO	1080	Yes	10∼150	[79] (2015)
Intermediate coupled model of ENSO (ICM) [51,52]	SPG and adjoint-based method	13 275	No	×	[47] (2017)
		8850	No	×	[48] (2019)
Three-dimensional thermohaline circulation model [54]	SPG and adjoint-based method	256	No	×	[30] (2016)
Quasi-geostrophic model	SQP [38] and adjoint-based method	2400	No	×	[49] (2008)
	COBYLA [65]	2400	Yes	30∼250	[64] (2017)
Shallow-water model	SPG and adjoint-based method	2.4 × 10^5^	No	×	[33] (2013)
ROMS [55]	SPG and adjoint-based method	∼1.71 × 10^7^	No	×	[39] (2018)
		∼1.24 × 10^7^	No	×	[50] (2016)
MM5 [56]	SPG and adjoint-based method	∼7.11 × 10^4^ ∼9.54 × 10^4^ ∼1.03 × 10^5^	No	×	[34] (2009)
		∼1.13 × 10^5^	No	×	[35] (2012)
	PCA-based GA	24024	Yes	100	[80] (2017)
WRF [57]	SPG and adjoint-based method	∼3.5 × 10^5^ ∼1.9 × 10^5^ ∼6.7 × 10^5^	No	×	[58,59] (2017, 2011)
Viscous Burgers equation	SPG and adjoint-free method	101	Yes	58	[40] (2010)
		101	Yes	56	[63] (2016)
GRAPES model [61]	SPG and adjoint-free method	23040	Yes	72	[61] (2009)
Lund-Potsdam-Jena model [77]	Differential evolution	240	No	×	[70–72] (2013, 2014, 2018)

Of course, it should be mentioned that the adjoint-based method for calculating the CNOP has three shortcomings. (i) The adjoint-based method can only deal with the smooth optimization problem in which the gradient of the objective function exists. For the non-smooth optimization problem without gradient, the adjoint-based method cannot be used. (ii) The adjoint models for many atmosphere and ocean models have not yet been developed and the adjoint method is thus unusable for these models. (iii) As the numerical model develops, the current model becomes very complicated, which causes the dimension of the optimization problem to become very large. The adjoint model thus needs massive storage space to save the basic state during each iteration. As such, the optimization time becomes very long. All of these create the challenges of data storage and timeliness. To overcome these disadvantages, different optimization strategies have been proposed to compute the CNOP, which will be reviewed in the following subsections.

### Adjoint-free optimization method

The adjoint-free optimization method includes the gradient definition-based method, the ensemble-based method with derivatives and the linear approximation method without derivatives. The former two are the optimization methods that need to calculate the gradient of the objective function. But the gradient is obtained by the numerical derivative or the ensemble run of the non-linear model rather than the adjoint model. The latter is a direct search optimization method without gradient computation.

In particular, ref. [60] employed the numerical derivative to obtain the gradient of the objective function and then calculated the CNOP within the medium-complexity Zebiak–Cane model (ZC model, [53]). Although this kind of gradient definition-based method is adjoint-free, it needs to perform a large number of non-linear model integrals, which has a huge computational cost. This method, therefore, may not be suitable for the complicated atmosphere and ocean model.

In addition, ref. [40] developed an ensemble-based method to compute the CNOP. The method calculates the approximate value of the gradient through building a statistical model ($ H\! $, see Equation (4)) between a limited number of ensemble perturbations $x{\rm{'}}$ that are linearly independent or orthogonal and their corresponding prediction increments $y'\!:$

(4) \begin{equation*}y'\! =\! {\rm{\ }}Hx'.\end{equation*}

A localization procedure has been used to obtain the above equation to filter out the long-distance correlations between the initial perturbations and the corresponding prediction increments on model grids. According to the statistical model, the gradient of the cost function can be easily computed by the following equation:

(5) \begin{equation*}{\nabla _{x'}}J\!\! \left( {x'} \right) = \ - 2{J\!}^2\!\left( {x'} \right){H^{\rm{T}}}y'.\end{equation*}

After obtaining the gradient information, the traditional optimization algorithms such as SPG or SQP could be used. Subsequently, refs. [40] and [61] applied this method to solve the CNOP in the theoretical Burgers function model with a 101-dimension cost function and a Global/Regional Assimilation and Prediction System (GRAPES) with more than 10^5^ dimension state variables.

Based on an idea similar to that of the above method, ref. [62] proposed a singular vector decomposition (SVD)-based ensemble method to reduce the dimension of the optimization problem to calculate the CNOP. The ensemble-based algorithm was applied to compute the CNOP for the ZC model with 1080 dimensional state variables. The optimal solutions obtained by the algorithm were acceptable and feasible and were close to those obtained by the adjoint-based method. In addition, as the localization procedure used by ref. [40] is very expensive due to its huge memory usage and repeated calculations, ref. [63] improved the localization implementation scheme and formulated a two-step (i.e. prediction–correction strategy) ensemble-based method to calculate the CNOP, which can save central processing unit (CPU) time and memory. Their numerical experiments were based on the three-level (L3) quasi-geostrophic (QG) global spectral model with a T21 truncation model and showed that the computation cost was indeed reduced.

For the linear approximation method without derivatives, ref. [64] first applied principal component analysis (PCA) to reduce the dimension of the optimization problem. After the dimension was decreased, the constrained optimization by linear approximation (COBYLA, [65]) algorithm was used to calculate the optimization solution. Ref. [64] used the QG model to test the method. They reduced the dimension of the optimization problem from 2400 to 10. Then, the COBYLA direct search optimization algorithm was employed to solve the optimization problem. However, they pointed out that, although this method is adjoint-free, it is much slower than the adjoint-based SQP algorithm, which means that it would cost more CPU time.

### Intelligent optimization method

The intelligent optimization method is gradient-free to directly capture the optimal value of the optimization problem. This type of method was thus also employed to compute the CNOP. For example, the genetic algorithm (GA, [66]) and particle swarm optimization (PSO, [67]) have been used to obtain the CNOP for models with different degrees of freedom, such as the theoretical Lorenz model and the 2D Ikeda model [41,68,69]. The larger dimensional (240-dimension) optimization problem [70–72] was solved to determine the CNOP related to parameters using the differential evolution (DE, [73]) algorithm. The DE algorithm is well compared to the traditional optimization algorithm [74,75]. In addition, ref. [76] used a cooperative coevolution-based particle swarm optimization (CCPSO) algorithm to solve the CNOP with a 270-dimension optimization problem in the ZC model to save the computational cost. The numerical results for the CNOP was similar to those of the adjoint-based method, and better than some other intelligent optimization algorithms.

**Figure 2.**
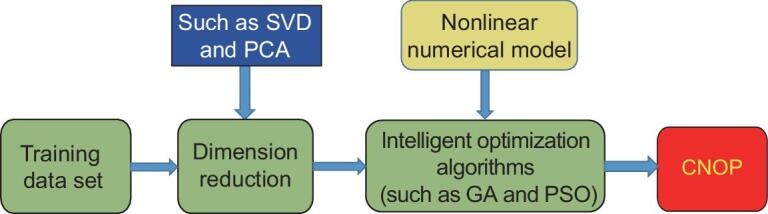
Framework for calculating the CNOP using the intelligent optimization algorithms with the dimension reduction method.

For the high-dimensional optimization problem, the intelligent optimization algorithms may fail to deliver the CNOP and its pattern. The descending dimension idea was introduced to obtain the CNOP using the intelligent optimization algorithms (Fig. 2) [78]. For example, ref. [79] used the PCA to transform the high-dimensional problem into the low-dimensional one. The original dimension of 1080 was reduced to the low dimensions of from 10 to 150. They found that the obtained CNOP was similar to that obtained by the adjoint-based method. Moreover, ref. [80] extended this idea, and applied the PCA-based GA method to solve the CNOP in the MM5 model. The original dimension of 24 024 was reduced to approximately 100. The GA also well compared with the adjoint-based method. The above results imply that the intelligent optimization method is a potential tool to obtain the CNOP for higher-dimensional optimization problems in the atmosphere and ocean science studies.

### Unconstrained optimization method

The CNOP was solved for a constrained optimization problem (COP). A natural question arises: could the COP be transformed into an unconstrained optimization problem (UOP)? Ref. [81] transformed *n*-dimension COP into (*n*–1)-dimension UOP with polar coordinates according to the theorem proved by ref. [82] that the CNOP-I is always located on the boundary of the constraint condition. The method was used to compute the CNOP for a terrestrial ecosystem model and performed well. Ref. [83] applied a penalty function (non-linear least-squares formulation) method and transformed the COP into the UOP. The CNOP was also compared using the T21L3 QG model for different constrained problems. The above methods supply a different view of obtaining the CNOP.

### Comparison of the CNOP-Is obtained by different approaches with the ZC model

To investigate the differences among CNOPs obtained by the four kinds of approaches mentioned above, we calculated the CNOPs related to initial conditions (CNOP-Is) using different methods based on the ZC model. It is worth pointing out that the adjoint-based approach, adjoint-free approach, intelligence optimization approach and unconstrained optimization approach used to calculate the CNOP-Is were based on references [46,62,79,81], respectively.

In the ZC model, two physical variables—the sea surface temperature anomaly (SSTA) and the thermocline height anomaly (THA)—were considered. The SSTA and THA were denoted by $TA$ and $HA$, respectively. In our calculation, the optimization time period was set to 9 months, with the initial optimization time of October. The objective function $J$ in Equation (3) is defined as $J\ = \sqrt {\mathop \sum \limits_{i,j} {{( {{w_1}T\!A{{( \tau )}_{i, j}}} )}^2\!}} $, where ${w_1} = {( {2.0^\circ C} )^{ - 1\!}} $, $\tau = 9$ months and $( {i,\ j} )$ represents the index of the grid cell. The constraint condition is set as ${C_\delta } = \{ {( {T\!A( 0 ),\ H\!A( 0 )} ){\rm{|}}}$${\sqrt {\mathop \sum \limits_{i, j} [{{( {{w_1}T\!A{{( 0 )}_{i, j}}} )}^2} + {{( {{w_2}H\!A{{( 0 )}_{i, j}}} )}^2}]} \le 1.0} \}$, where ${w_2} = {( {50\ m} )^{ - 1}}$. Notably, the PCA is used in the adjoint-free approach and the intelligence method. According to the suggestions in [62] and [79], 70 PCs and 40 PCs were used in our computation for the adjoint-free approach and the intelligence method, respectively. The more detailed settings for the ZC model and the optimization calculation can be found in [62,79].

**Figure 3.**
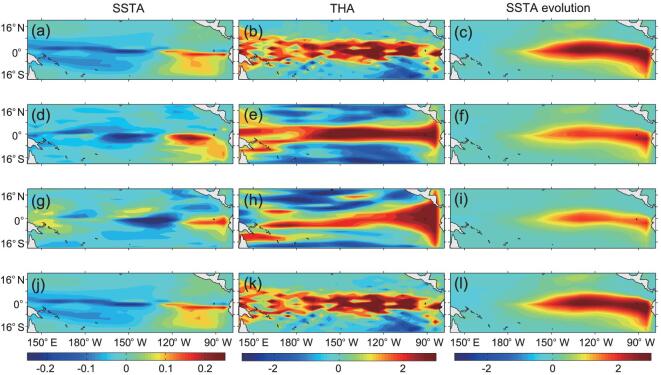
The SSTA (°C, left panel) and THA (m, middle panel) components of the CNOP-Is obtained by the adjoint-based approach (a)–(c), adjoint-free approach (d)–(f), intelligence optimization approach (g)–(i) and unconstrained optimization approach (j)–(l) based on references [46,62,79,81] within the ZC model. The CNOP-Is are calculated at the initial optimization time of October. The right panel shows the evolution of the SSTA after 9 months.

Figure 3 shows the CNOP-Is obtained by the different approaches. Taking a glance at the figure, we can see that the dominant features of the CNOP-Is for different computational methods are roughly similar. The spatial patterns of the SSTA components exhibit a zonal dipole with positive anomalies in the eastern tropical Pacific and negative anomalies in the central tropical Pacific. The THA components show a uniform deepening mode over the entire equatorial Pacific. But some detail differences among the CNOP-Is exist. For example, the THA anomalies for the adjoint-based approach and unconstrained method have more small-scale features, whereas the adjoint-free method and the intelligence method give the smoother THA pattern. Besides, the evolutions of the CNOP-Is show that the El Niño events caused by the CNOP-Is for the adjoint-based approach and unconstrained method are stronger than those for the adjoint-free method and the intelligence method. This may be because only the several dominant PC modes were used in the latter two methods.

Of course, it should be mentioned that, for each kind of method, the above results are only from one algorithm. It is worth investigating the validity for other algorithms described in [40,64,80,83], for example, in the future.

## SUMMARY AND DISCUSSION

The CNOP method has become a useful tool in the predictability studies of atmosphere and ocean, but also in several important issues in fluid mechanics. This paper reviewed the development of the CNOP approach and its computation. The approach was first proposed for exploring the non-linear optimal initial perturbation that has the largest non-linear evolution. In recent years, the approach has been extended to investigate the optimal perturbation in the model parameters, the model tendencies and the boundary conditions. Because this review mainly focused on CNOP computation, the applications of the method were not illustrated here. Interested readers are encouraged to refer to the review papers [84–86].

The key step of the CNOP method is how to obtain solutions to the optimal perturbation. Various optimization methods have been developed to calculate the CNOP. Here, we reviewed the four kinds of commonly used methods, including the adjoint-based method, the adjoint-free method, the intelligent optimization method and the unconstrained optimization method. All of these methods have their own advantages and disadvantages. The adjoint-based method has a relatively fast convergence rate because the increasing direction of the objective function can be obtained by the adjoint model. The adjoint code, however, is often unavailable for complex atmosphere and ocean models. The adjoint-free method and the intelligent optimization method do not need the adjoint code but they need to perform a great number of non-linear model integrals, which will cost many computational resources and much time. The unconstrained optimization method can reduce the dimension of the optimization method but the reduction is so limited that this method may be not able to significantly save the computational load. As an example, the CNOP-Is calculated by different kinds of methods with the ZC model were compared. The results show that the dominant structures of the CNOP-Is for different methods are similar, although some differences in details exist. This implies that all the tested methods are valid.

As atmospheric and oceanic models are developed, they become more and more complex and the model resolution becomes finer, which causes some challenges for CNOP calculation. Both the dimension of the optimization problem and the computational cost become very large. In this situation, on the one hand, we need to develop efficient dimensionality reduction technology to reduce the problem dimension according to the considered physical problem itself, as is done in the adjoint-free ensemble method and the intelligent optimization method. On the other hand, more importantly, it is necessary to develop new optimization methods. For example, with the progress of computer technology, supercomputers have quickly developed and computing powers have significantly improved. Efficiently adopting high-performance computing resources to develop high-efficiency parallel optimization methods is an important way to solve the CNOP calculation problem. Of course, this requires the full cooperation of the experts from fields of mathematics, computer science, and atmosphere and ocean science. Through this cooperation, it is expected that CNOP-related optimization calculation can be well resolved in the future.
